# *Six2* Plays an Intrinsic Role in Regulating Proliferation of Mesenchymal Cells in the Developing Palate

**DOI:** 10.3389/fphys.2017.00955

**Published:** 2017-11-23

**Authors:** Dennis O. Okello, Paul P. R. Iyyanar, William M. Kulyk, Tara M. Smith, Scott Lozanoff, Shaoping Ji, Adil J. Nazarali

**Affiliations:** ^1^Laboratory of Molecular Cell Biology, Neuroscience Research Cluster, College of Pharmacy and Nutrition, University of Saskatchewan, Saskatoon, SK, Canada; ^2^Department of Anatomy and Cell Biology, College of Medicine, University of Saskatchewan, Saskatoon, SK, Canada; ^3^Med-life Discoveries LP, Saskatoon, SK, Canada; ^4^Department of Anatomy, Biochemistry and Physiology, John A. Burns School of Medicine, University of Hawaii, Honolulu, HI, United States; ^5^Department of Biochemistry and Molecular Biology, Medical School, Henan University, Kaifeng, China

**Keywords:** *Six2*, palate, *Hoxa2*, craniofacial development, cell proliferation, *Cyclin D1*

## Abstract

Cleft palate is a common congenital abnormality that results from defective secondary palate (SP) formation. The *Sine oculis-related homeobox 2* (*Six2*) gene has been linked to abnormalities of craniofacial and kidney development. Our current study examined, for the first time, the specific role of *Six2* in embryonic mouse SP development. *Six2* mRNA and protein expression were identified in the palatal shelves from embryonic days (E)12.5 to E15.5, with peak levels during early stages of palatal shelf outgrowth. Immunohistochemical staining (IHC) showed that Six2 protein is abundant throughout the mesenchyme in the oral half of each palatal shelf, whereas there is a pronounced decline in Six2 expression by mesenchyme cells in the nasal half of the palatal shelf by stages E14.5–15.5. An opposite pattern was observed in the surface epithelium of the palatal shelf. Six2 expression was prominent at all stages in the epithelial cell layer located on the nasal side of each palatal shelf but absent from the epithelium located on the oral side of the palatal shelf. *Six2* is a putative downstream target of transcription factor *Hoxa2* and we previously demonstrated that *Hoxa2* plays an intrinsic role in embryonic palate formation. We therefore investigated whether *Six2* expression was altered in the developing SP of *Hoxa2* null mice. Reverse transcriptase PCR and Western blot analyses revealed that *Six2* mRNA and protein levels were upregulated in *Hoxa2*^−/−^ palatal shelves at stages E12.5–14.5. Moreover, the domain of Six2 protein expression in the palatal mesenchyme of *Hoxa2*^−/−^ embryos was expanded to include the entire nasal half of the palatal shelf in addition to the oral half. The palatal shelves of *Hoxa2*^−/−^ embryos displayed a higher density of proliferating, Ki-67 positive palatal mesenchyme cells, as well as a higher density of Six2/Ki-67 double-positive cells. Furthermore, *Hoxa2*^−/−^ palatal mesenchyme cells in culture displayed both increased proliferation and elevated *Cyclin D1* expression relative to wild-type cultures. Conversely, siRNA-mediated *Six2* knockdown restored proliferation and *Cyclin D1* expression in *Hoxa2*^−/−^ palatal mesenchyme cultures to near wild-type levels. Our findings demonstrate that *Six2* functions downstream of *Hoxa2* as a positive regulator of mesenchymal cell proliferation during SP development.

## Introduction

Cleft palate is a common congenital malformation in humans, with a complex etiology (Vanderas, 1987). The palate separates the nasal and oral cavities, allowing for proper respiration, feeding and phonation. Both genetic and environmental factors have been implicated in the causation of cleft palate (Dixon et al., 2011). However, the molecular mechanisms involved in the pathogenesis of cleft palate are poorly understood.

Mouse secondary palate (SP) development begins around embryonic day (E) 11.5, with the emergence of paired palatal shelf outgrowths from the maxillary prominences. From E12.0–13.5, the palatal shelves grow vertically downwards on either side of the developing tongue. At E14.0, the tongue depresses, allowing the two palatal shelves to re-orient horizontally above the tongue. The elevated palatal shelves grow horizontally toward each other, establishing contact to form the midline epithelial seam (MES) at E14.5. The MES degrades by E15.5, creating a confluent SP. The SP then fuses anteriorly with the primary palate, a derivative of the converged medial nasal processes, to complete the formation of the roof of the oral cavity by E16.5 (Ferguson, 1988; Kaufman, 1992). In addition, mesenchymal cells located in the anterior portion of the SP ossify to form the palatine bone. Disruptions in the growth, elevation or fusion of the palatal shelves can lead to congenital cleft palate defects (Ferguson, 1988; Gritli-Linde, 2007; Smith et al., 2013).

*Sine oculis-related homeobox 2* (*Six2*) is a member of the vertebrate *Six* gene family which encode homeobox transcription factors homologous to the *Drosophila* Sine oculis protein (Kawakami et al., 2000). *Six* family genes have been reported to promote cell proliferation and survival during embryogenesis (Kawakami et al., 2000). *Six2* is expressed primarily in the cranial base, midface, facial prominences, first pharyngeal arch, and in the urogenital region of the developing embryo (Fogelgren et al., 2008). *Six2* null mice die at birth exhibiting renal hypoplasia (Self et al., 2006) and a shorter cranial base (He et al., 2010). In these mice, chondrocyte differentiation in the cranial base is abnormal, with decreased cell proliferation and increased terminal differentiation leading to premature fusion of the cranial base (He et al., 2010).

Downregulation of *Six2* by microRNAs miR-181b or miR-181c inhibits cell proliferation and promotes apoptosis in metanephric kidney mesenchymal cells *in vitro* (Lyu et al., 2013; Lv et al., 2014). Transcription factor Zeb1, a marker of epithelial-mesenchymal transitions during embryogenesis and cancer metastasis, regulates cell proliferation in metanephric mesenchymal cells by binding to the *Six2* promoter and upregulating its expression (Gu et al., 2016). Additionally, *Six2* promotes metastasis of breast cancer cells by repressing E-cadherin expression via mechanisms involving miR-200b downregulation, Zeb 2 upregulation, and *E-cadherin* promoter methylation (Wang et al., 2014).

In the radiation-induced mouse mutant *brachyrrhine* (*Br/Br*), prenatal deficiency of *Six2* leads to frontonasal dysplasia, cleft palate (Singh et al., 1998; McBratney et al., 2003) and renal hypoplasia (McBratney et al., 2003; Fogelgren et al., 2008, 2009). Moreover, investigations have linked *Six2* deletion in humans to an autosomal dominant frontonasal dysplasia syndrome that has similarities to the murine *Br* mutant phenotype (Hufnagel et al., 2016).

Deletions of the *Hoxa2* gene in mice also lead to cleft palate defects, together with altered morphogenesis of second pharyngeal arch structures (Rijli et al., 1993 and Gendron-Maguire et al., 1993). Investigations in our laboratory have previously demonstrated that *Hoxa2* is expressed intrinsically within the palatal shelves of wild-type mouse embryos (Nazarali et al., 2000), where it inhibits proliferation of the palatal mesenchyme cells (Smith et al., 2009). The possibility that *Six2* plays a specific role in SP development has not been previously examined. In our present study we demonstrate, for the first time, that *Six2* is expressed intrinsically in both the palatal shelf mesenchyme and palatal shelf epithelium of wild-type mouse embryos, and further show that *Six2* mRNA and protein are upregulated in the palatal shelves of *Hoxa2*^−/−^ mice. In addition, we provide evidence that *Six2* functions downstream of *Hoxa2* to regulate mesenchymal cell proliferation within the developing secondary palate.

## Materials and methods

### *Hoxa2* transgenic mice

*Hoxa2*^+/−^ mice were maintained by backcrossing to C57BL/6J wild-type mice and the heterozygous mice were intercrossed to generate *Hoxa2*^+/+^ and *Hoxa2*^−/−^ embryos for analysis in this study (Smith et al., 2009). Pregnant mice were sacrificed by isoflurane inhalation followed by cervical dislocation. Embryos were staged according to Kaufman (1992) and were considered E0 on the day the vaginal plug was found. Genotypes were confirmed by PCR analyses as described in Gendron-Maguire et al. (1993). The protocol for the use of animals was approved by the University of Saskatchewan's Animal Research Ethics Board and adhered to Canadian Council on Animal Care guidelines for humane animal use.

### Immunohistochemistry

Embryos were harvested from timed-pregnant mice and the heads were fixed with 4% paraformaldehyde in phosphate buffered saline, pH 7.4 (PBS) for 24 h. Embryo heads were placed in 30% sucrose in PBS for at least 24 h, followed by embedding in optimal cutting temperature compound (OCT; Tissue-Tek®) and serially sectioned at 10 μm thickness. Histological sections taken anterior or posterior to the first molar tooth bud were designated as anterior and posterior palate sections, respectively, and sections taken at the plane of the first molar tooth bud were designated as middle palate sections (Welsh and O'Brien, 2009; Smith et al., 2013). Tissue sections were placed on 0.5% gelatin-coated glass slides, air-dried at room temperature for at least 2 h, rehydrated for 30 min in PBS, and blocked for 30 min at room temperature in PBS containing 4% skim milk and 0.1% Triton X-100. Sections were then incubated overnight at 4°C with primary antibody diluted in PBS. The antibodies used were: Six2 rabbit polyclonal antibody (Proteintech®; 1:500 dilution), E-cadherin rat monoclonal antibody (Sigma; 1:200 dilution) and Ki-67 rat monoclonal antibody (Affymetrix eBioscience®; 1:100 dilution). Sections were rinsed twice for 5 min in PBS followed by incubation with secondary antibody for 1 h at room temperature (anti-IgG Alexa fluor 488 antibody, 1:200 dilution or anti-IgG Alexa fluor 594, 1:400 dilution; Molecular Probes®). Finally, sections were rinsed twice in PBS and mounted in ProLong® Gold antifade reagent with DAPI (Molecular Probes®).

### RNA isolation and reverse transcription

Total RNA was isolated from excised palatal shelves of wild-type and *Hoxa2*^−/−^ embryos at stages E12.5, E13.5, E14.5, and E15.5 using Aurum Total RNA mini kit (BioRad®) as per the manufacturer's protocol. First-strand cDNA synthesis was performed using SuperScript reverse transcriptase (Invitrogen®) with 1 μg of total RNA as per the manufacturer's protocol (Smith et al., 2009).

### Quantitative real time PCR (qRT-PCR)

Gene expression analysis was performed on palatal shelf cDNA samples as described in our previous study (Thangaraj et al., 2017). Briefly, a TaqMan® gene expression assay was used for qRT-PCR analysis of relative *Six2* mRNA expression levels. All qRT-PCR reactions were performed using 25 ng of template cDNA, TaqMan Universal Master Mix, FAM-labeled TaqMan Gene Expression assay Mm03003557_S1 for *Six*2 (Applied Biosystems®), and VIC-labeled endogenous control TaqMan assay for β*-actin* (Applied Biosystems® assay 4352341E). *Cyclin D1* expression was quantified by SYBR Green assay using forward primer 5′-ACCCTGACACCAATCTCCTC-3′ and reverse primer 5′-AAGCGGTCCAGGTAGTTCAT-3′. All reactions were run in biological replicates of 5. Thermocycling parameters were: 2 min at 50°C, 10 min at 95°C, followed by 40 cycles of 95°C for 15 s and 60°C for 70 s. The C_T_ values obtained were analyzed using the 2^−ΔΔ*CT*^ method to determine the relative expression of target genes in wild-type and *Hoxa2* null samples.

### Droplet digital PCR (ddPCR)

To independently confirm the results of our qRT-PCR analyses, we also performed ddPCR gene expression analyses on palatal shelf cDNA samples, following established protocols (Hindson et al., 2013). Briefly, oil-emulsified PCR reaction mixtures containing palatal shelf cDNA were amplified in 96-well plates on a Bio-Rad Tetrad 2 Peltier Thermal Cycler under the following conditions: 95°C for 10 min then 40 cycles of 95°C for 15 s and 60°C for 1 min (2.5°C/s ramp rate) with a final 10 min hold at 98°C. After amplification, the plates were transferred to a Bio-Rad QX 100 Droplet Reader, which aspirated oil-emulsified PCR products from each well and counted numbers of FAM-positive and VIC-positive droplets, sampling at 100 kHz. Discrimination between droplets containing amplified target (positives) from those which did not (negatives) was achieved by applying a global fluorescence amplitude threshold. Gene transcript concentrations for each palatal RNA sample were calculated using dedicated ddPCR Poisson distribution computational modeling software (Bio-Rad®). We employed the same *Six2* and β*-actin* TaqMan assays for our ddPCR analyses as described in our qRT-PCR protocol.

### Western blot analysis

Palatal shelves were dissected from wild-type or *Hoxa*2^−/−^ embryos and homogenized in RIPA Buffer (150 mM NaCl, 10 mM Tris, 0.1%-SDS, 1% Triton X-100, 1% deoxycholate, 5 mM EDTA) supplemented with a protease inhibitor cocktail (Sigma®) as previously described (Brown and Nazarali, 2010). Sample aliquots containing equal total protein were loaded onto 10% polyacrylamide-SDS gels. Following electrophoresis, the proteins were transferred to Immunoblot PVDF membranes (Bio-Rad®). The membranes were blocked overnight at 4°C in PBS containing 4% skim milk, followed by incubation for 1 h at room temperature with rabbit polyclonal Six2 primary antibody (Proteintech®; diluted 1:2,000 in PBS containing 4% skim milk). This was followed by four 15 min washes in PBST (PBS containing 0.08% Tween-20) and incubation for 1 h at room temperature with horseradish peroxidase-conjugated anti-rabbit secondary antibody (Bio-Rad®; diluted 1:3,000 in PBS containing 4% skim milk). After four 15 min washes in PBST, the membranes were incubated with Clarity® Western ECL substrate (Bio-Rad®) and the signal detected using a SYNGENE® image analyzer. As a control for equal protein loading, the membranes were subsequently washed overnight at 4°C in PBS, followed by incubation with anti-β-tubulin (Developmental Studies Hybridoma Bank) at 1:2,000 dilution for 1 h at room temperature. Subsequently, membranes were washed with PBST, incubated with anti-mouse IgG horseradish peroxidase conjugate (Bio-Rad®; 1:2,000 dilution), and processed for chemiluminescence protein detection as described above. After imaging, semi-quantitative densitometry was performed using AlphaView® software to generate an integrated density value for each Six2 protein band, which was normalized to the β-tubulin density value from the same sample. Four separate Western blots, each having both wild-type and *Hoxa2*^−/−^ samples from E12.5, E13.5, E14.5, E15.5 palatal shelves, were performed. For each blot, the normalized Six2 expression values in the various samples (wild-type and *Hoxa2*^−/−^ palates at E12.5, E13.5, E14.5, E15.5 stages) were compared to the value of the E12.5 wild-type sample on the same blot, which was arbitrarily assigned a relative expression level of 1. A total of four blots (*n* = 4) were analyzed using two-way ANOVA to compare Six2 protein expression in wild-type and *Hoxa2*^−/−^ samples.

### Culture of mouse embryonic palate mesenchymal (MEPM) cells

Primary cultures of MEPM cells were established as previously described (Iwata et al., 2012; Iyyanar and Nazarali, 2017). Briefly, palatal shelves from E13.5 embryos were aseptically micro-dissected and placed in Hank's balanced salt solution. The palatal shelves were then incubated with 0.25% trypsin/EDTA in Ca^+^/Mg^++^-free PBS for 20 min at 37°C, briefly triturated, and passed through a 70 μm cell strainer (BD Falcon®) to generate a dissociated mesenchymal cell suspension. Trypsin action was terminated by adding complete DMEM/F-12 medium (Dulbecco's Modified Eagle's Medium/Ham's medium F12 [1:1] supplemented with 10% fetal bovine serum, 1% antibiotic/antimycotic solution [Sigma®], and L-glutamate) to the cell suspension. The cells were plated on poly-D-lysine coated plates and cultured at 37°C in 5% CO_2_ incubator.

### siRNA treatment and cell proliferation analysis

A pre-designed *Six2* siRNA (Invitrogen® Silencer Select assay s73795) and a negative control siRNA (Invitrogen®) were utilized. MEPM cells (5 × 10^3^) were plated in a 96-well plate until they reached 60–80% confluency. Aliquots of 50 nM siRNA were mixed with Lipofectamine 3000 (Invitrogen®) according to the manufacturer's protocol. The siRNA-Lipofectamine complex (10 μl) was added to each well of MEPM cells containing 100 μl of serum free medium. Following incubation at 37°C for 12 h, the transfection medium was replaced with fresh medium. MEPM cells were analyzed for cellular DNA content after 48 h from the time of transfection using the CyQUANT NF cell proliferation assay kit (Life Technologies®) following the manufacturer's protocol. Briefly, the transfected MEPM cells were incubated with 1X dye binding solution at 37°C for 45 min in the dark. Fluorescence was measured with a BioTek® microplate reader at 485 nm excitation and 530 nm emission wavelengths.

### Cell density determination

Following IHC staining of histological sections, the number of Ki-67 positive mesenchymal cells in the palatal shelf were counted manually using Image J software and this number was divided by the total cross-sectional area (mm^2^) of the palatal mesenchyme. Additionally, the numbers of Ki-67 positive mesenchyme cells in the nasal vs. oral halves of the sectioned palatal shelves were counted and divided by their respective areas (mm^2^); *n* = 4.

### Statistical analyses

All statistical analyses and graph construction were performed using GraphPad® Prism 5.0 software. The Western blot, gene expression, and cell count data were evaluated using two-way analysis of variance (ANOVA) followed by Bonferroni *post-hoc* tests.

## Results

### *Six2* mRNA and protein are expressed in the developing palate and upregulated in *Hoxa2^−/−^* mice

**Figure 1:**
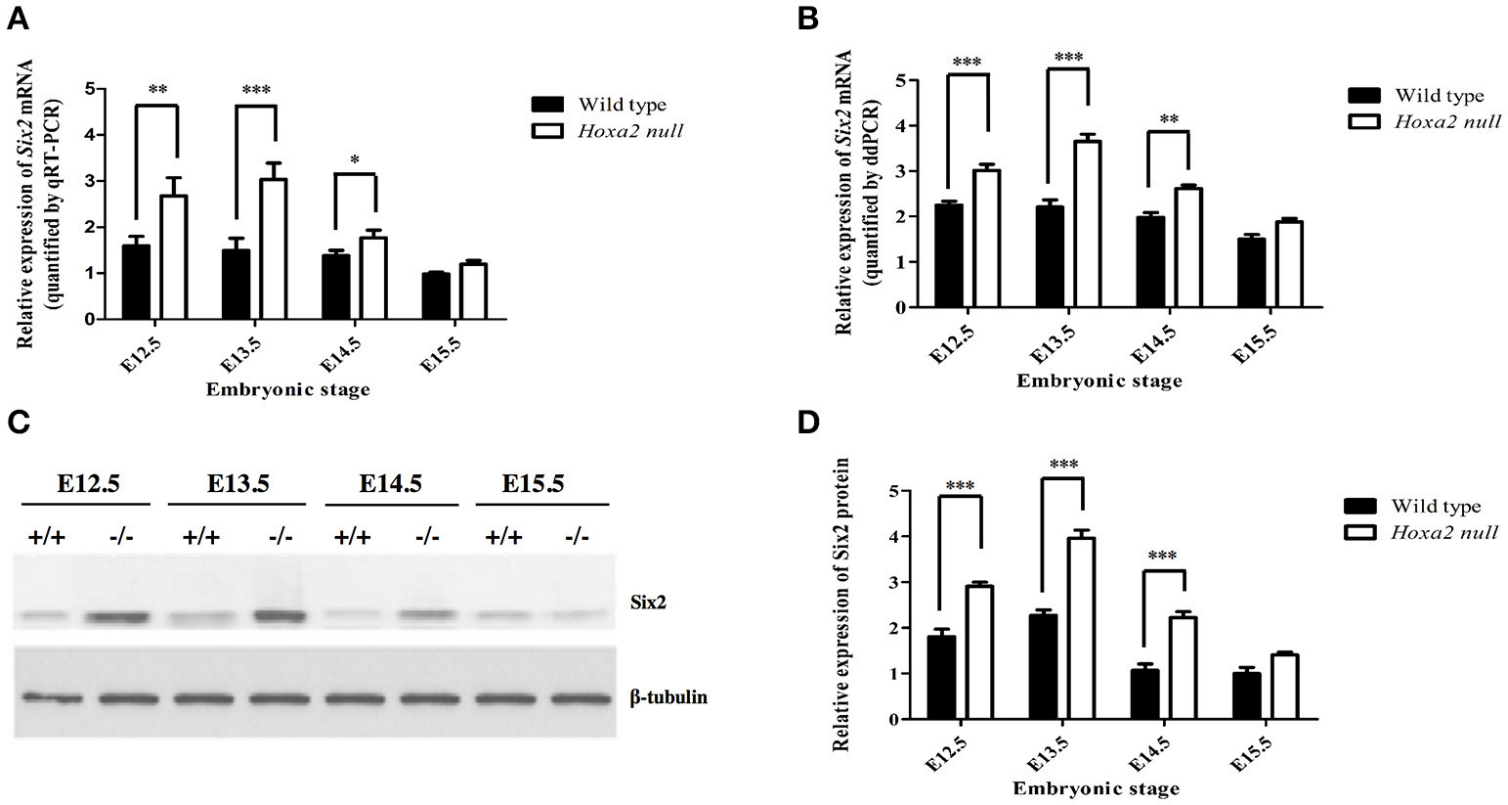
Temporal changes in *Six2* mRNA **(A,B)** and Six2 protein **(C,D)** levels in the palatal shelves of wild-type and *Hoxa2*^−/−^ mouse embryos at developmental stages E12.5-E15.5. **(A)** qRT-PCR analysis of relative levels of *Six2* mRNA expression in the palatal shelves of wild-type and *Hoxa2*^−/−^ mouse embryos at developmental stages E12.5-E15.5. The *Six2* mRNA expression values are normalized against expression levels of the β*-actin* reference gene (*n* = 5 biological replicates). Note that *Six2* mRNA levels were significantly higher in *Hoxa2*^−/−^ palatal shelves compared to wild-type from stages E12.5 to E14.5. **(B)** Droplet digital PCR (ddPCR) quantification of relative *Six2* mRNA levels in palatal shelves of wild-type and *Hoxa2*^−/−^ embryos at stages E12.5–E15.5 (*n* = 5 biological replicates). Western blots **(C)** and corresponding densitometric measurements **(D)** of temporal changes in Six2 protein levels in wild-type and *Hoxa2*^−/−^ palatal shelves during palatogenesis (*n* = 4 biological replicates). Note that Six2 protein is significantly upregulated in palatal shelves of *Hoxa2*^−/−^ embryos from E12.5 to E14.5. Two-way ANOVA followed by Bonferroni *post-hoc* test was performed for each analysis. Bars represent mean ± SEM. ^*^p < 0.05, ^**^p < 0.01, ^***^p < 0.001.

Our qRT-PCR analyses revealed that *Six2* mRNA is expressed in the developing palatal shelves of wild-type mouse embryos from E12.5 to E15.5, with highest expression at E12.5 and E13.5 (Figure 1A). The palatal shelves of *Hoxa2*^−/−^ null embryos showed a significant upregulation of *Six2* mRNA levels relative to wild-type palates at stages E12.5 to E14.5 (Figure 1A). These trends were confirmed on independent biological samples using the ddPCR technique as an alternate method to quantify *Six2* gene transcript levels (Figure 1B). Consistent with the *Six2* mRNA expression profiles, Western blot analysis revealed that Six2 protein is present in the developing palatal shelves from E12.5 to E15.5, with peak expression at E13.5 in wild-type embryos (Figures 1C,D). At stages E12.5 to E14.5, Six2 protein levels were significantly higher in *Hoxa2*^−/−^ palatal shelves compared to wild-type palatal shelves (Figure 1D). These results demonstrate that *Six2* is expressed intrinsically in the developing palate and is negatively regulated by *Hoxa2* during palatogenesis.

### Six2 protein distribution in the developing secondary palate exhibits temporal and spatial variations

**Figure 2:**
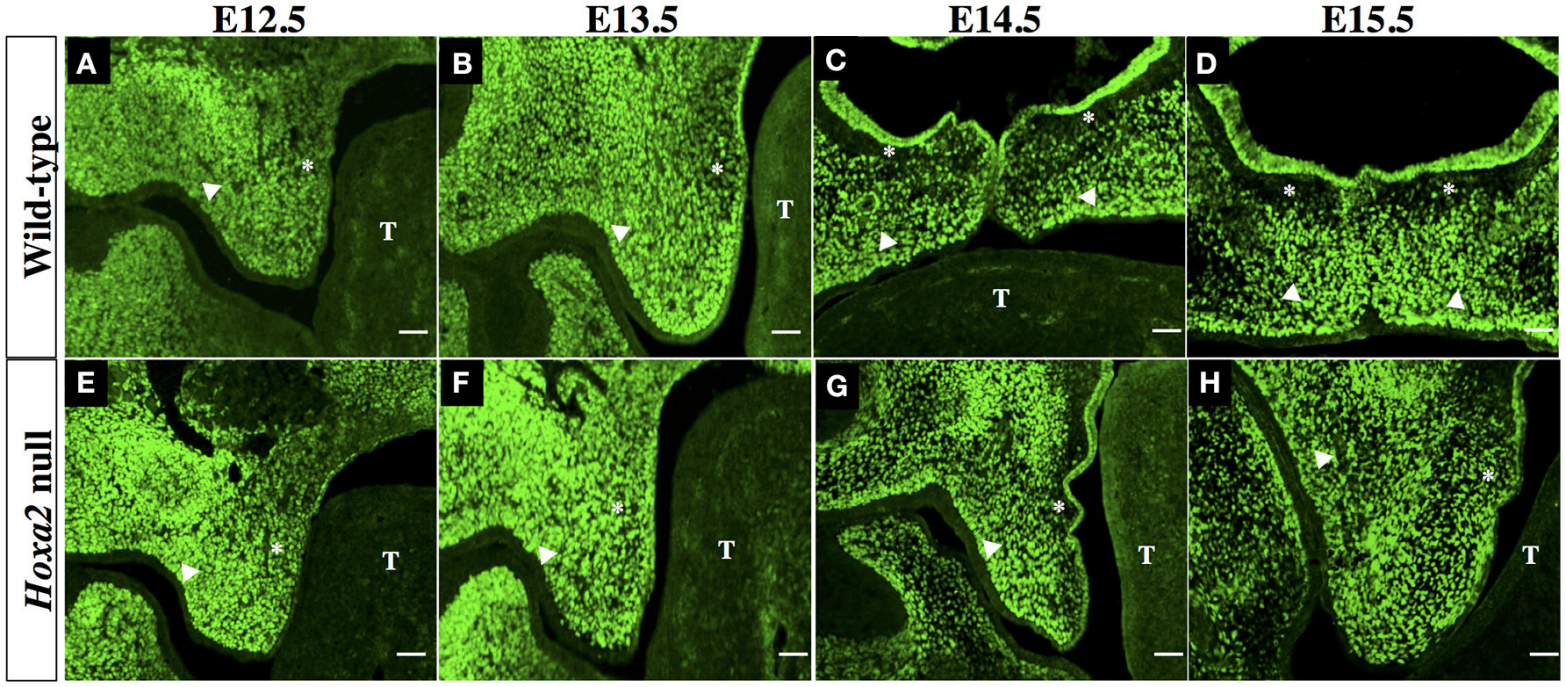
Immunohistochemical (IHC) analysis of Six2 localization in the palatal shelves of wild-type **(A–D)** and *Hoxa2* null embryos **(E–H)** at developmental stages E12.5 **(A,E)**, E13.5 **(B,F)**, E14.5 **(C,G)**, and E15.5 **(D,H)**. Photomicrographs are mid-coronal sections, and are representative of a minimum of 5 biological replicates per stage. In each photograph, the asterisk indicates the nasal half of the palatal shelf and the arrow head indicates the oral half of the palatal shelf. T, identifies the developing tongue. Six2 immunostaining in the mesenchyme of wild-type palatal shelves appeared most intense at stages E12.5 **(A)** and E13.5 **(B)**, and was uniformly distributed throughout the palatal shelf at these early stages. However, by stages E14.5 **(C)** to E15.5 **(D)** there was a pronounced decline in Six2 expression by a layer of mesenchyme cells in the nasal half of the palatal shelf lying immediately beneath the palatal epithelium. In *Hoxa2*^−/−^ embryos **(E–H)**, Six2 immunostaining persisted throughout the palatal mesenchyme within the nasal half as well as the oral half of the palatal shelf at all stages from E12.5-15.5 **(E–H)**. Note that the palatal shelves of *Hoxa2* null embryos fail to elevate and remain oriented vertically alongside the tongue **(G,H)**. Scale bar, 50 μm.

We next examined the spatial distribution of Six2 protein in the developing SP. IHC analyses of mid-coronal sections of heads from wild-type mouse embryos revealed abundant Six2 protein expression in the mesenchyme of the palatal shelves from stages E12.5 to E15.5 (Figures 2A–D). The intensity of Six2 immunostaining in the palatal shelf mesenchyme of wild-type embryos appeared higher at earlier stages of palatogenesis (E12.5–E13.5) (Figures 2A,B) compared to later stages (E14.5–E15.5) (Figures 2C,D). At stages E12.5 and E13.5, prior to palatal shelf elevation, Six2 protein was observed throughout the palatal mesenchyme in both the prospective “nasal half” of the palatal shelf (located nearest the tongue at these pre-elevation stages) as well as in the “oral half” of the palatal shelf (located furthest from the tongue) (Figures 2A,B). However, by E14.5 to E15.5, after the wild-type palatal shelves have reoriented to a horizontal position above the tongue, there was a conspicuous loss of Six2 immunostaining within a layer of palatal mesenchyme located in the nasal half of the palatal shelf, immediately subjacent to the surface palatal epithelium (Figures 2C,D). In contrast, Six2 protein expression persisted throughout the palatal mesenchyme in the oral half of the palatal shelf in wild type embryos (Figures 2C,D).

The palatal shelves of *Hoxa2* null embryos, unlike those of wild-type embryos, fail to elevate and instead remain oriented vertically downward on either side of the developing tongue (Figures 2E–H). Within these *Hoxa2*^−/−^ palatal shelves, Six2 protein expression persisted through stage E15.5 in palatal mesenchyme cells of both the nasal half of the palatal shelf (positioned nearest the tongue) as well as the oral half of the palatal shelf (located furthest from the tongue) (Figures 2E–H). Therefore, in comparison to wild-type embryos, the loss of *Hoxa2* function expands the spatial domain of Six2 expression within the palatal mesenchyme at stages E14.5–E15.5 (compare Figures 2A–D to Figures 2E–H).

**Figure 3:**
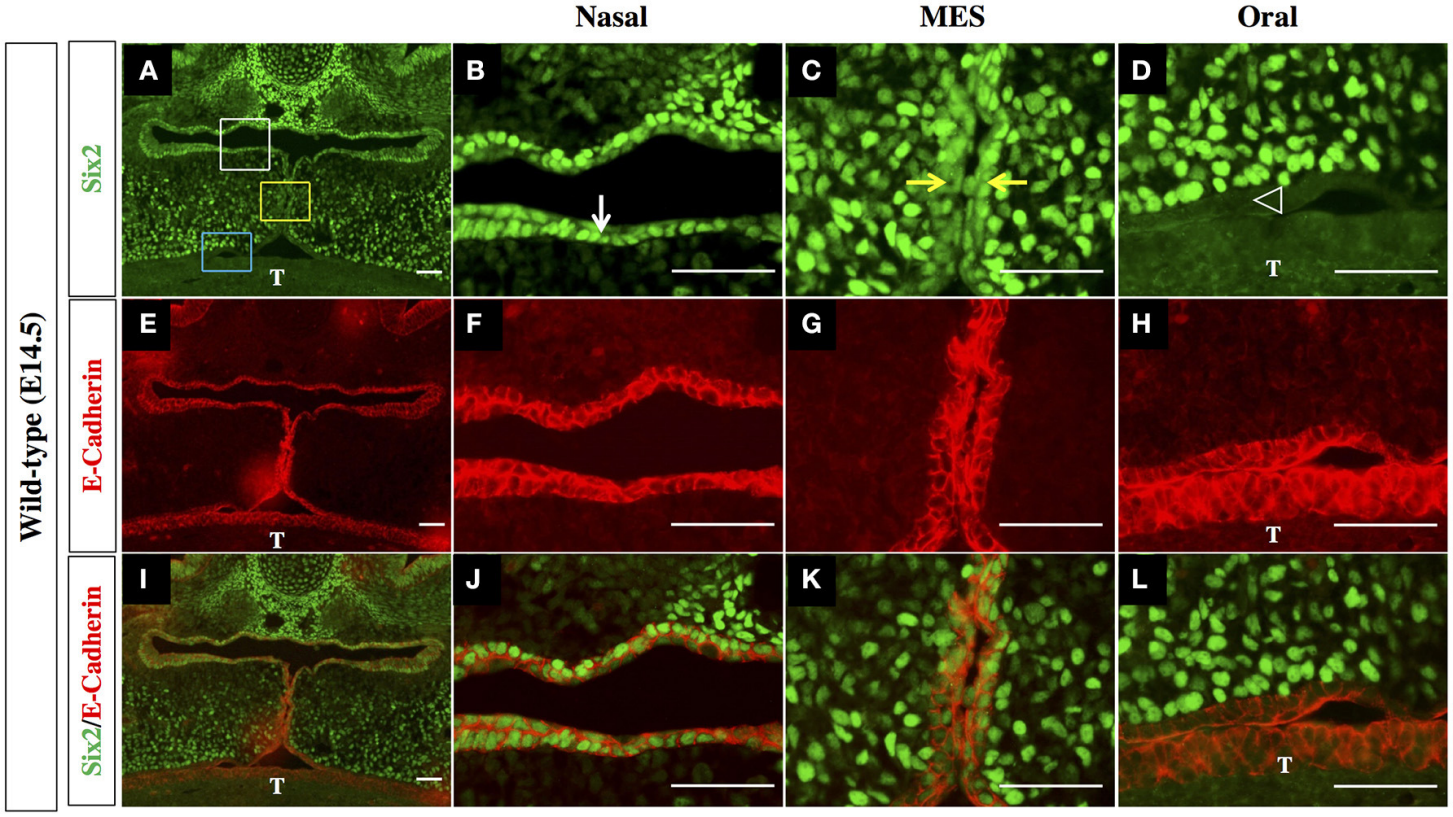
Six2 protein is differentially expressed by epithelium on the nasal side vs. the oral side of the palatal shelf in wild-type mice. IHC staining of mid-coronal sections of wild-type palatal shelves at E14.5 for Six2 **(A–D)** and E-Cadherin **(E–H)**. Panels I-L are the Six2 immunofluorescence images overlaid with the E-Cadherin immunofluorescence images to identify cells co-expressing the two proteins. Six2 protein expression is prominent in epithelial cells located on nasal side of the palatal shelf (marked by the white rectangle in panel **(A)**, and by the white arrow in panel **(B)**, where it is co-expressed with the epithelial marker E-cadherin **(E,F,I,J)**. Six2 is also expressed in epithelial cells of the MES (marked by yellow rectangle in panel A, and by yellow arrows in panel **(C)**, which is the point of contact between the apical tips of the two elevated palatal shelves. In contrast, Six2 protein is absent from the epithelium on the oral side of the palatal shelf [marked by the blue rectangle in panel **(A)** and by position of the arrowhead in panel **(D)**], which expresses E-cadherin alone **(E,H,I,L)**. MES, midline epithelial seam; T, tongue. Scale bars, 50 μm. The IHC staining images shown are representative of palatal sections from a minimum of 5 embryos.

**Figure 4:**
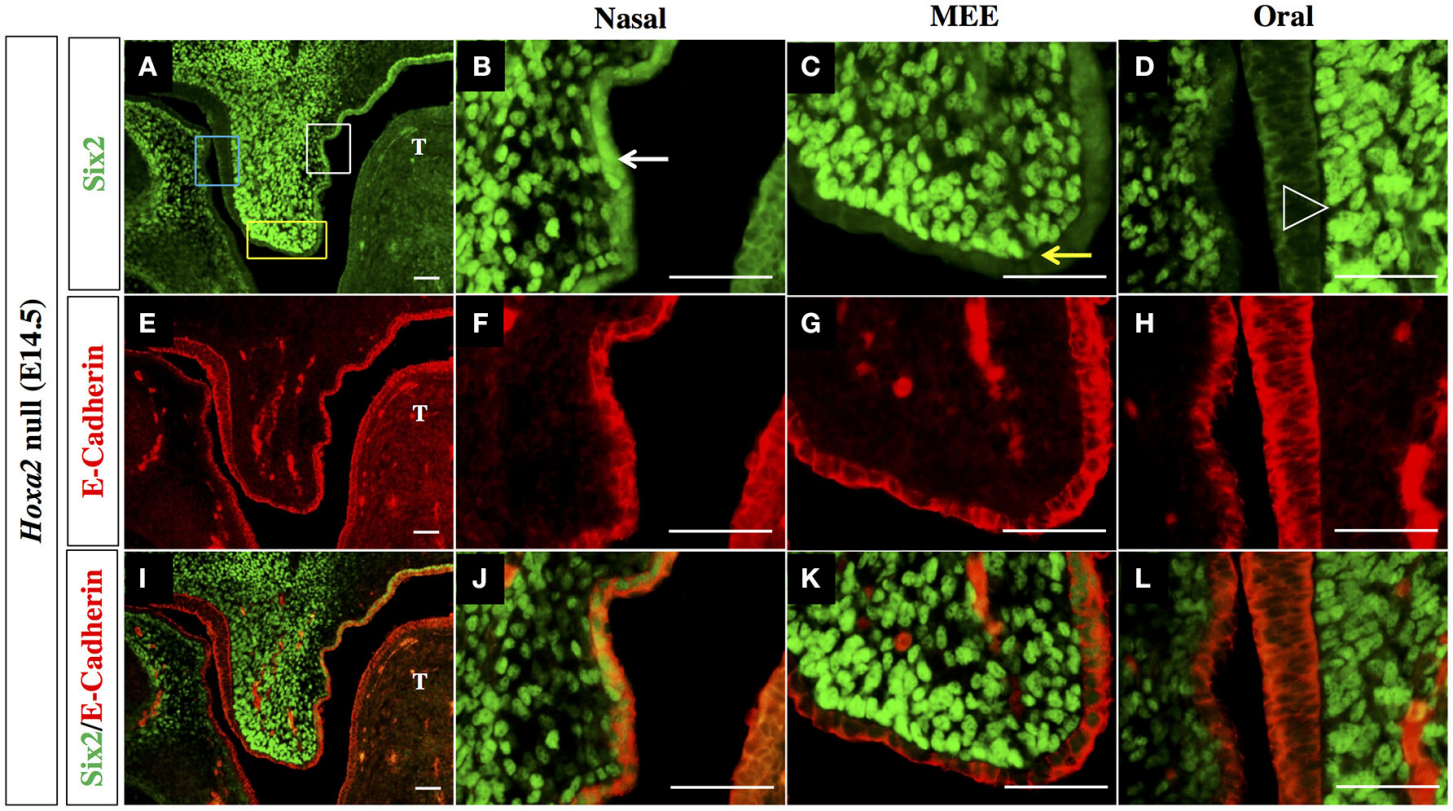
Six2 protein is exclusively expressed in epithelium on the nasal side of the palatal shelf in *Hoxa2*^−/−^ mice. IHC staining of mid-coronal sections of *Hoxa2* null palatal shelves at E14.5 for Six2 **(A–D)**, E-Cadherin **(E–H)**, and overlays of the Six2 and E-Cadherin immunofluorescence images **(I–L)**. Expression of Six2 protein within the palatal epithelium is restricted to epithelial cells located on the nasal side of the palatal shelf (marked by white rectangle in panel **(A)**, and by white arrow in the higher magnification image **(B)**. Six2 is absent from epithelial cells located on the oral side of the palatal shelf (marked by blue rectangle in **A**, and by position of arrowhead in higher magnification image **D**). Within *Hoxa2*^−/−^ embryos, Six2 is also absent from cells of the medial edge epithelium (MEE) which is located at the apical tip of the palatal shelf (marked by yellow rectangle in **(A)**, and by yellow arrow in higher magnification image **(C)**. Because palatal shelves of *Hoxa2*^−/−^ fail to elevate and make contact, they do not form an MES. The entire surface epithelium of *Hoxa2*^−/−^ palatal shelves (on both its nasal and oral sides, as well as within the MEE) expresses E-cadherin **(E–H, I–L)**. MEE, medial edge epithelium; T, tongue. Scale bars, 50 μm. The IHC staining images shown are representative of palatal sections from a minimum of 5 embryos.

The outer epithelial cell layer that coats the nasal and oral surfaces of the palatal shelf displayed a strikingly different pattern of Six2 protein distribution. In wild-type palatal shelves, Six2 immunostaining was prominent in the surface epithelium located on the nasal side of the palatal shelf at both pre-elevation and post-elevation stages (Figures 2A–D; Figures 3A,B). Conversely, Six2 protein was undetectable in the surface epithelium located on the oral side of the palatal shelf (Figures 2A–D, 3A,D). The pattern of Six2 expression within the palatal epithelium was largely unaffected by loss of *Hoxa2* function. In *Hoxa2*^−/−^ embryos, like wild-type embryos, Six2 expression was prominent in epithelial cells located on the nasal side of the palatal shelf (facing the tongue), with little or no Six2 protein detectable in the epithelium covering the oral side of the palatal shelf (located furthest from the tongue) (Figures 2E–H, 4A,B,D). These findings were confirmed by co-staining palatal sections from wild-type (Figure 3) and *Hoxa2*^−/−^ embryos (Figure 4) with an antibody for E-cadherin, a characteristic epithelial marker (Figures 3E–L, 4E–L). Examination of the palatal sections at high magnification revealed that the surface epithelium on the nasal side of the palatal shelf co-expressed both Six2 and E-cadherin proteins in wild-type embryos (Figures 3B,F,J) as well as in *Hoxa2*^−/−^ mutants (Figures 4B,F,J). By contrast, epithelial cells on the oral side of the palatal shelf were positive for E-cadherin alone in both wild-type (Figures 3D,H,L) and *Hoxa2*^−/−^ embryos (Figures 4D,H,L).

Interestingly, in the palatal shelves of E14.5 wild-type embryos, we also observed prominent expression of Six2 together with E-cadherin within cells of the MES, the point of contact/adhesion between the apical tips of the two horizontally elevated palatal shelves (Figures 3C,G,K). However, Six2 immunostaining was only faintly visible in medial edge epithelium (MEE) cells located at the apical tips of the *Hoxa2*^−/−^ palatal shelves, which fail to elevate and make midline contact (Figures 4C,G,K).

### Expression of the Ki-67 cell proliferation marker is enhanced in the *Hoxa2*^−/−^ palatal shelves

**Figure 5:**
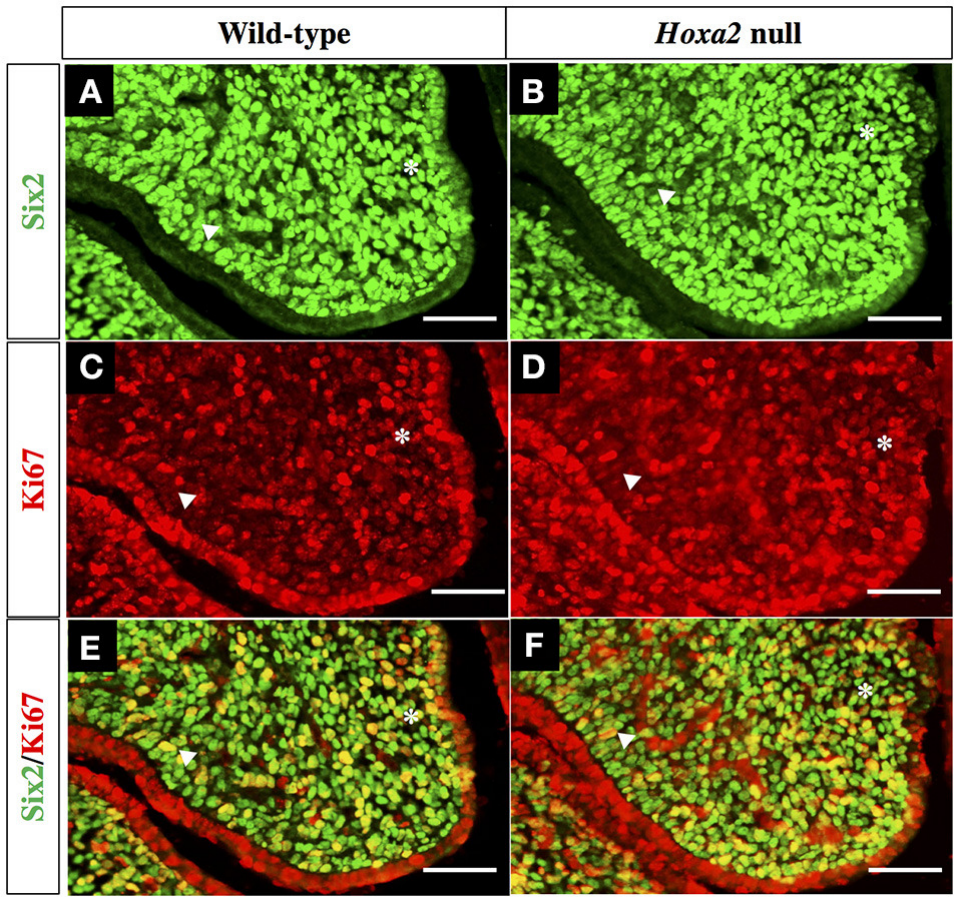
Double IHC staining of wild-type and *Hoxa2*^−/−^ palatal shelves with antibodies for Six2 as well as Ki-67, a marker of proliferating cells. Representative coronal sections from the middle region of E13.5 wild-type palatal shelves **(A,C,E)** and *Hoxa2*^−/−^ palatal shelves **(B,D,F)**. Green fluorescence in panels **(A,B)** identifies Six2 expressing cells, whereas red fluorescence in panels **(C,D)** identifies all cells expressing proliferation marker Ki-67. Panels **(E,F)** are overlays of the Six2 **(A,B)** and Ki-67 **(C,D)** immunofluorescence images to identify cells that co-expressed Six2 together with Ki-67. Both wild-type **(E)** and *Hoxa2*^−/−^ **(F)** palatal shelves contained numerous Six2/Ki-67 double-positive mesenchyme cells, which appear yellow in these image overlays. Six2/Ki-67 double-positive cells appear higher in the *Hoxa2*^−/−^ palate. Asterisk indicates the nasal half of the palatal shelf and the arrowhead indicates the oral half of the palatal shelf. Scale bars, 50 μm. The IHC staining images shown are representative of palatal sections from a minimum of four wild-type and four *Hoxa2* null embryos.

To explore the relationships between *Six2, Hoxa2* and cell proliferation during SP development, we performed double immunofluorescence staining on histological sections from the anterior, middle, and posterior regions of E13.5 wild-type and *Hoxa2* null palatal shelves using Six2 antibody in combination with an antibody for Ki-67, a nuclear protein expressed exclusively in proliferating cells (Figure 5).

**Figure 6:**
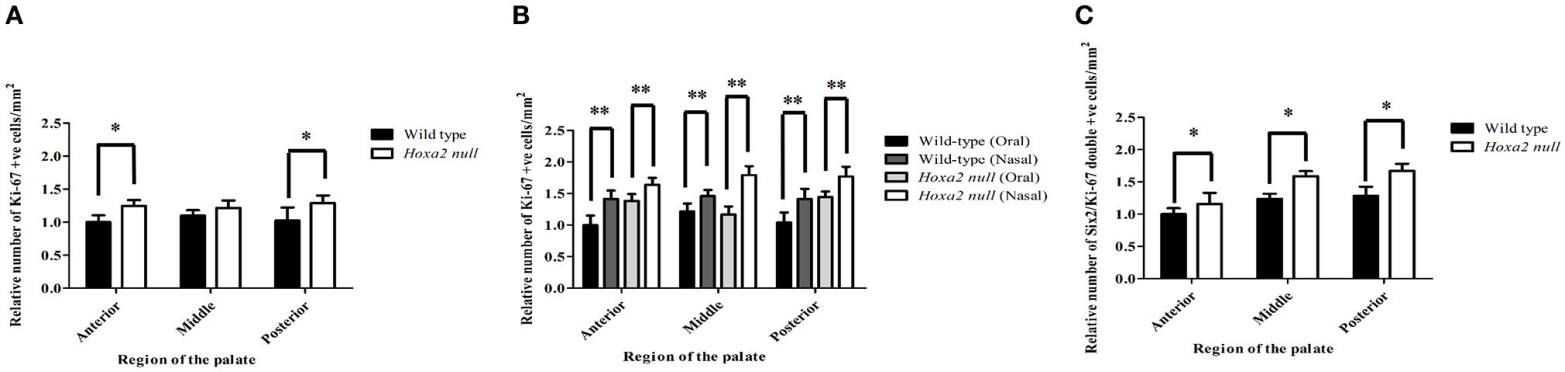
The densities of proliferating, Ki-67 positive and Six2/Ki-67 double-positive mesenchyme cells are elevated in *Hoxa2*^−/−^ palatal shelves compared to wild-type. **(A)** Relative numbers of Ki-67 positive palatal mesenchyme cells per mm^2^ in coronal sections taken from anterior, middle and posterior regions of E13.5 wild-type and *Hoxa2*^−/−^ palatal shelves. In the anterior and posterior regions, the density of proliferating palatal mesenchyme cells was significantly higher in *Hoxa2*^−/−^ palatal shelves compared to wild-type. **(B)** In both wild-type and *Hoxa2*^−/−^ palatal shelves, the density of proliferating palatal mesenchyme cells was consistently higher in the nasal half vs. the oral half of the palatal shelf. **(C)** Relative numbers of Six2-positive mesenchyme cells that co-expressed Ki-67 (i.e., were actively proliferating) in the anterior, middle and posterior regions of *Hoxa2*^−/−^ palatal shelves compared to wild-type. Bar graphs represent mean ± SEM; *n* = 4 biological replicates. ^*^p < 0.05, ^**^p < 0.01.

We observed that the mesenchyme of both wild-type and *Hoxa2*^−/−^ palatal shelves contained large numbers of proliferating, Ki-67 positive cells (Figures 5C,D). Cell counts performed on the immunostained sections revealed that the number of Ki-67 positive palatal mesenchyme cells per unit area was significantly higher in *Hoxa2*^−/−^ palatal shelves compared to wild-type in both the anterior and posterior regions of the palate (Figure 6A). This trend was also observed in the middle region of the palate, although the difference there was not statistically significant. Interestingly, in all three regions along the A-P axis of the palate (anterior, middle, and posterior), the density of proliferating Ki-67 positive mesenchyme cells was significantly higher in the nasal half of the palatal shelf compared to its oral half (Figure 6B). This was the case for both wild-type as well as *Hoxa2* null embryos (Figure 6B).

Many Six2-expressing palatal mesenchyme cells in both wild-type and *Hoxa2* null embryos were actively proliferating, as evidenced by Ki-67 co-expression (Figures 5E, F). Cell counts revealed that the numbers of these Six2/Ki-67 double-positive palatal mesenchyme cells per unit area were significantly higher in *Hoxa2*^−/−^ mice compared to wild-type in all three regions along the A-P axis of the palate (anterior, middle, and posterior) (Figure 6C).

### *Six2* knockdown reduces cell proliferation and *Cyclin D1* expression in MEPM palatal mesenchyme cell cultures

**Figure 7:**
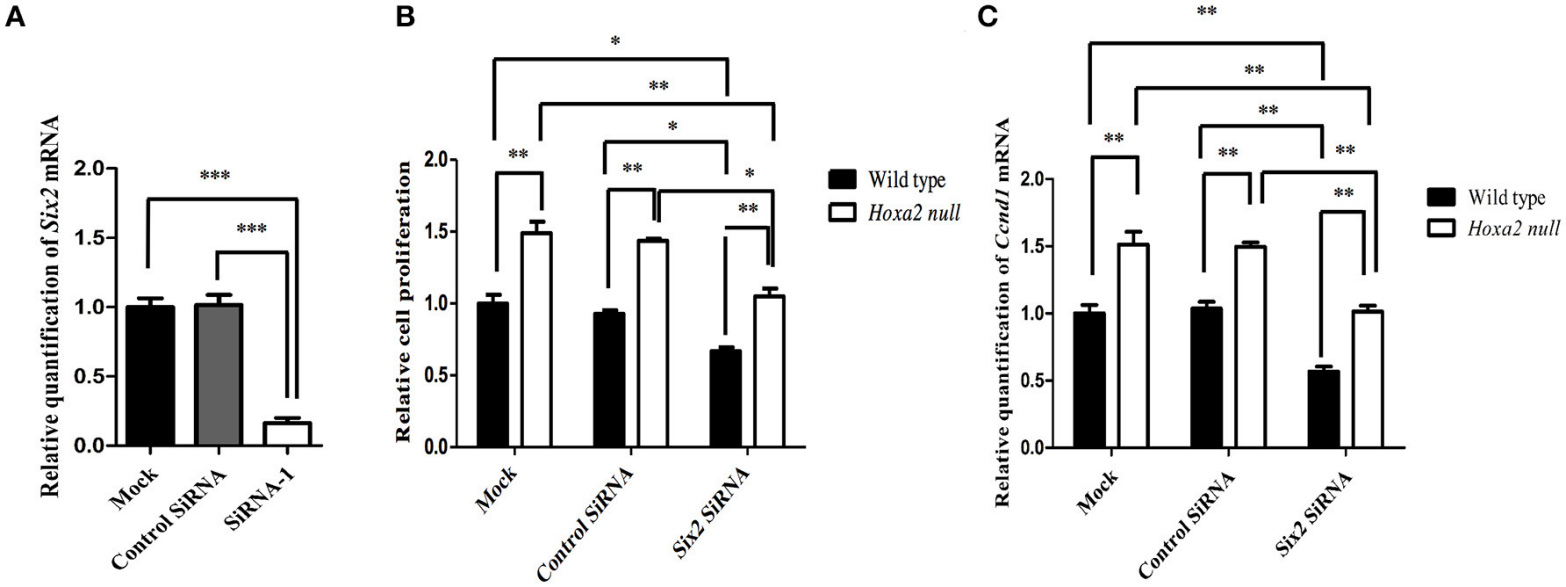
*Six2* knockdown in wild-type and *Hoxa2*^−/−^ mouse embryonic palate mesenchyme (MEPM) cultures reduces cell proliferation and *Cyclin D1* expression. **(A)** Treatment of MEPM cultures with *Six2* siRNA decreased *Six2* mRNA expression by 90% compared to cultures administered either a negative control siRNA or siRNA delivery-vehicle alone (mock treatment). **(B)** *Six2* siRNA treatment significantly decreased cell proliferation in both wild-type MEPM and *Hoxa2*^−/−^ MEPM cultures and also reduced expression of *Cyclin D1* mRNA **(C)**. Note that levels of cell proliferation **(B)** and *Cyclin D1* expression **(C)** in *Hoxa2*^−/−^ MEPM cultures were consistently higher than those of parallel wild-type cultures in all three treatment groups. Also, *Six2* knockdown restored cell proliferation and *Cyclin D1* expression in *Hoxa2*^−/−^ MEPM cultures to levels approximating those of control wild-type MEPM cultures. Bars represent mean ± SEM; *n* = 4 biological replicates. ^*^p < 0.05, ^**^p < 0.01, ^***^p < 0.001.

We next investigated whether *Six2* is a positive regulator of cell proliferation in the palatal mesenchyme and whether upregulation of *Six2* is responsible for the increased cell proliferation in *Hoxa2*^−/−^ palatal mesenchyme. Primary cultures of MEPM cells were transfected with siRNA targeting *Six2* mRNA to determine the effects of *Six2* knockdown on proliferation of palatal mesenchyme cells *in vitro*. As shown in Figure 7A, treatment with *Six2* siRNA resulted in ~90% reduction in *Six2* mRNA expression in MEPM cultures when compared to cultures administered either siRNA delivery vehicle alone (mock treatment) or a non-targeting negative control siRNA. Importantly, siRNA-mediated *Six2* knockdown decreased cell proliferation in both wild-type and *Hoxa2*^−/−^ MEPM cultures (Figure 7B) and also reduced mRNA levels of *Cyclin D1*, a cell cycle regulator (Figure 7C). In the *Hoxa2*^−/−^ MEPM cultures, *Six2* knockdown restored both cell proliferation and *Cyclin D1* expression down to levels approximating those of wild-type control MEPM cultures treated with either siRNA delivery vehicle alone or the negative control siRNA (Figures 7B,C).

## Discussion

Previous studies have established that the *Six2* gene is expressed within multiple regions and tissues of vertebrate embryos, including the developing head, pharyngeal arches, and kidneys (Oliver et al., 1995; Kutejova et al., 2005; Fogelgren et al., 2008). Furthermore, *Six2* mutations are linked to embryonic craniofacial and renal malformations (Singh et al., 1998; McBratney et al., 2003; Self et al., 2006; Fogelgren et al., 2008, 2009), which appear to result in part from reduced cell proliferation during organogenesis (Self et al., 2006; He et al., 2010). Our present study demonstrates, for the first time, that *Six2* transcripts and Six2 protein are expressed endogenously within the palatal shelves of wild-type mouse embryos throughout the period of normal SP formation. Moreover, we found that Six2 expression within the palatal primordia is both temporally modulated and spatially heterogeneous.

Our gene expression and Western blot data indicate that *Six2* mRNA and protein levels are quantitatively highest during the early stages of SP formation (E12.5–13.5), when the palatal shelves emerge as paired outgrowths of the two maxillary prominences and grow vertically downwards on either side of the developing tongue. *Six2* mRNA and protein expression persist, albeit at quantitatively lower levels, within the palatal processes during the subsequent phases of palatal shelf elevation, contact, and fusion (at E14.5–E15.5) which culminate in the separation of the oral and nasal cavities. The lateral palatine processes undergo progressive enlargement during early phases of palatogenesis, suggesting the possibility that the Six2 transcription factor may assist in promoting palatal shelf tissue growth. Supporting this possibility, we have demonstrated that siRNA-mediated knockdown of *Six2* mRNA expression in cultures of palatal shelf mesenchyme cells resulted in reduced mesenchymal cell proliferation as well as reduced mRNA levels of the cell cycle regulator, *Cyclin D1*.

In addition to the temporal variation in *Six2* expression levels during SP development, our IHC analyses revealed intriguing heterogeneity in its spatial distribution within the mesenchyme and epithelium of the growing palatal shelves. Within the palatal mesenchyme of wild-type embryos, Six2 protein was expressed uniformly throughout mesenchymal cells located in the prospective oral half of the palate, at stages both prior to and following palatal shelf elevation. By contrast, palatal shelf elevation in wild-type embryos was accompanied by a marked loss of Six2 expression by a band of palatal mesenchyme cells located in the nasal half of the palatal shelf. Interestingly, a somewhat converse pattern of Six2 distribution was observed in the surface epithelium of the palatal shelves, such that Six2 expression was prominent within epithelial cells located on the nasal side of each palatal shelf, whereas little or no Six2 protein was detectable within epithelial cells on the oral side of the palatal shelves. Several other genes have been previously shown to exhibit differential expression along the oro-nasal (O-N) axis of the developing SP. Like *Six2*, the *Fgf10* (Rice et al., 2004), *Foxf1* (Lan and Jiang, 2009; Xu et al., 2016), *Gli1* (Han et al., 2009; Lan and Jiang, 2009), *Osr2* (Lan and Jiang, 2009), and *Ptch1* (Lan and Jiang, 2009) genes are all predominantly expressed in the oral half of the palatal mesenchyme (reviewed in Bush and Jiang, 2012). Conversely, *Pax9* exhibits higher expression in mesenchyme in the nasal half of palatal shelf (Lan et al., 2004; Zhou et al., 2013). It remains to be explored whether the localized expression of *Six2* along the O-N axis in the palatal mesenchyme or the palatal epithelium either regulates or is regulated by, the domains of expression of any of these other genes. Alternatively, the loss of Six2 expression by a population of mesenchymal cells in the nasal half of the palatal shelf might be a consequence of the onset of osteogenic differentiation in this location, since bone formation during SP development is confined to the nasal half of the palatal shelves (Han et al., 2009; Baek et al., 2011). A number of transcription factors and signaling molecules also demonstrate gradations in their expression levels along the A-P (anterior-posterior) axis of the developing SP including *Msx1, Bmp4, Bmp2, Shh, Spry2, Fgf10, Fgf7, Shox2, Meox2, Tbx22*, and *Barx1* (reviewed in Bush and Jiang, 2012; Smith et al., 2013). However, our study revealed no significant quantitative differences in Six2 expression levels between anterior, middle and posterior regions of the palate.

Our study also examined the relationship between *Hoxa2* function within the developing palatal shelves and the regulation of *Six2* expression therein. Our gene expression and Western blot analyses revealed that *Six2* mRNA and Six2 protein levels are significantly elevated in palatal shelves of *Hoxa2*^−/−^ mouse mutants, and our IHC data demonstrate that the domain of Six2 expression in *Hoxa2*-null palatal shelf mesenchyme is ectopically expanded to include the entire nasal half of the palatal shelf in addition to the oral half. These findings suggest that *Hoxa2* acts as a negative regulator of *Six2* expression within palatal shelf mesenchyme. Consistent with our observations, earlier studies by Kutejova et al. (2005, 2008) showed that the *Six2* gene is an immediate downstream target of transcription factor Hoxa2 in the second pharyngeal arch which, through negative regulation, confines Six2 expression to the more anterior first pharyngeal arch. Previous investigations in our own laboratory have shown that the *Hoxa2* gene is expressed endogenously in the epithelium and mesenchyme of the developing palatal shelves (Nazarali et al., 2000; Smith et al., 2009). However, unlike Six2, we have observed no conspicuous difference in Hoxa2 expression levels between the oral and nasal halves of the palate. Therefore, other genes in addition to *Hoxa2* must regulate *Six2* expression domains within the palatal mesenchyme of wild-type embryos to account for the greater Six2 protein abundance in the oral half of the palatal shelf vs. the nasal half. Moreover, within the outer epithelium layer of the palate, the spatial expression pattern of Six2 must be independent of Hoxa2 function, since Six2 protein remains confined to epithelial cells on the nasal side of the palatal shelf in *Hoxa2*^−/−^ mutants, as it is in wild-type embryos.

The palatal shelves originate from outgrowths of the maxillary prominence derivatives of pharyngeal arch 1. However, somewhat paradoxically, *Hoxa2* expression is normally absent from tissue of the first pharyngeal arch itself, and the loss of *Hoxa2* function in *Hoxa2*^−/−^ mutant mice leads to ectopic formation of arch 1 skeletal structures in place of arch 2 elements (Gendron-Maguire et al., 1993; Rijli et al., 1993). Studies from several labs have shown that *Hoxa2* null mice develop cleft palate defects *in vivo* (Gendron-Maguire et al., 1993; Rijli et al., 1993; Barrow and Capecchi, 1999). We have previously demonstrated that *Hoxa2* knockdown in whole palatal organ culture explants resulted in failure of the palatal shelves to fuse *ex vivo* (Smith et al., 2009). We have now extended those findings by showing that the cleft palate defects in *Hoxa2*^−/−^ palatal shelves are accompanied by elevated levels and spatially expanded expression of Six2 protein, as well as increased proliferation of the palatal mesenchyme cells. This suggests the possibility that *Six2* expression within the normally developing SP may positively regulate palatal mesenchyme cell proliferation and, furthermore, that the increased mesenchymal cell proliferation observed in *Hoxa2* null palatal mesenchyme may result from increased expression of endogenous *Six2*. This is supported by our observation that, *in vivo*, the numbers of palatal mesenchyme cells that co-express Six2 together with the cell proliferation marker Ki-67 are higher in *Hoxa2*^−/−^ palatal shelves than wild-type palatal shelves. Moreover, we demonstrated that treatment of wild-type palate mesenchymal cell cultures with *Six2* siRNA to knockdown *Six2* expression led to significant reductions in both cell proliferation and *Cyclin D1* mRNA levels. Indeed, whereas *Hoxa2*^−/−^ MEPM cultures otherwise displayed enhanced cell proliferation, *Six2* knockdown in the *Hoxa2* null cultures restored mesenchymal proliferation to wild-type levels.

From our findings, it appears likely that increased Six2 expression leading to a rise in the level of palatal mesenchyme proliferation is responsible, at least in part, for the generation of cleft palate defects in *Hoxa2*^−/−^ embryos. This is consistent with studies from other laboratories that have implicated increased mesenchymal proliferation as contributing to cleft palate formation in *Wnt5a*^−/−^ (He et al., 2008) and *Sprouty2*^−/−^ mice (Welsh et al., 2007; Matsumura et al., 2011), as well as in embryos expressing *Fgf8* ectopically in palatal mesenchyme (Wu et al., 2015). Apparently, rates of mesenchymal cell proliferation must be tightly regulated for normal growth, morphogenesis and elevation of the palatal shelves, with either increased or decreased proliferation of palatal mesenchyme potentially leading to cleft palate defects (reviewed in Smith et al., 2013).

In summary, our study is the first to specifically investigate the expression and cell proliferation function of *Six2* in the developing SP. We have demonstrated that *Six2* mRNA and protein exhibit dynamic temporal and spatial expression profiles within the mouse SP from embryonic stages E12.5 through to E15.5. We observed that Six2 protein is present within both the mesenchyme and epithelium of the developing SP; however, its spatial distribution within these two cell populations displays an intriguing pattern of complementarity along the O-N axis of the palatal shelf. Additionally, we demonstrated that *Six2* functions downstream of *Hoxa2*, which negatively regulates *Six2* expression. Finally, we provide new evidence supporting a role for *Six2* as a positive regulator of mesenchymal cell proliferation in the developing SP.

## Author contributions

AN conceived and coordinated the study, with assistance from WK. DO together with PI designed and performed experiments, analyzed the data, and prepared initial manuscript drafts. AN and WK extensively revised subsequent versions of the manuscript contents and interpretations of results. TS performed pilot experiments that laid the foundation for this investigation. SL was involved in the initial study design and critically reviewed the manuscript. SJ assisted with experimental design and data analysis. All authors approved the final manuscript version for submission.

### Conflict of interest statement

The authors declare that the research was conducted in the absence of any commercial or financial relationships that could be construed as a potential conflict of interest.

## Abbreviations

ddPCR: digital droplet PCR
E: embryonic day
IHC: immunohistochemical
MEE: medial edge epithelium
MEPM: mouse embryonic palatal mesenchyme
MES: midline epithelial seam
O-N: oro-nasal
qRT-PCR: quantitative real-time PCR
SP: secondary palate.
